# Beneficial Modulation of Lipid Mediator Biosynthesis in Innate Immune Cells by Antirheumatic *Tripterygium wilfordii* Glycosides

**DOI:** 10.3390/biom11050746

**Published:** 2021-05-17

**Authors:** Kehong Zhang, Simona Pace, Paul M. Jordan, Lukas K. Peltner, Alexander Weber, Dagmar Fischer, Robert K. Hofstetter, Xinchun Chen, Oliver Werz

**Affiliations:** 1 Department of Pharmaceutical/Medicinal Chemistry, Institute of Pharmacy, Friedrich-Schiller-University, Philosophenweg 14, 07743 Jena, Germany; kehong.zhang@uni-jena.de (K.Z.); simona.pace@uni-jena.de (S.P.); paul.jordan@uni-jena.de (P.M.J.); lukas.klaus.peltner@uni-jena.de (L.K.P.); robert.klaus.hofstetter@uni-jena.de (R.K.H.); 2 Guangdong Provincial Key Laboratory of Regional Immunity and Diseases, Department of Pathogen Biology, Shenzhen University School of Medicine, Shenzhen 518000, China; chenxinchun@szu.edu.cn; 3 Department of Chemistry and Pharmacy, Pharmaceutical Technology, Friedrich-Alexander-Universität Erlangen-Nürnberg, Cauerstrasse 4, 91058 Erlangen, Germany; alexander.christian.weber@fau.de (A.W.); dagmar.fischer@fau.de (D.F.)

**Keywords:** *Tripterygium wilfordii* glycosides, lipoxygenase, cyclooxygenase, inflammation, specialized pro-resolving mediators

## Abstract

*Tripterygium wilfordii* glycosides (TWG) is a traditional Chinese medicine with effectiveness against rheumatoid arthritis (RA), supported by numerous clinical trials. Lipid mediators (LM) are biomolecules produced from polyunsaturated fatty acids mainly by cyclooxygenases (COX) and lipoxygenases (LOX) in complex networks which regulate inflammation and immune responses and are strongly linked to RA. The mechanism by which TWG affects LM networks in RA treatment remains elusive. Employing LM metabololipidomics using ultra-performance liquid chromatography-tandem mass spectrometry revealed striking modulation of LM pathways by TWG in human monocyte-derived macrophage (MDM) phenotypes. In inflammatory M1-MDM, TWG (30 µg/mL) potently suppressed agonist-induced formation of 5-LOX products which was confirmed in human PMNL and traced back to direct inhibition of 5-LOX (IC_50_ = 2.9 µg/mL). TWG also efficiently blocked thromboxane formation in M1-MDM without inhibiting other prostanoids and COX enzymes. Importantly, in anti-inflammatory M2-MDM, TWG (30 µg/mL) induced pronounced formation of specialized pro-resolving mediators (SPM) and related 12/15-LOX-derived SPM precursors, without COX and 5-LOX activation. During MDM polarization, TWG (1 µg/mL) decreased the capacity to generate pro-inflammatory 5-LOX and COX products, cytokines and markers for M1 phenotypes. Together, suppression of pro-inflammatory LM but SPM induction may contribute to the antirheumatic properties of TWG.

## 1. Introduction

Lipid mediators (LM) encompass oxygenated polyunsaturated fatty acids (PUFA) which are involved in maintenance of normal hemostasis but also display significant roles in host defense as well as in pain, fever and in inflammation and its resolution [1,2,3]. They are mainly derived from arachidonic acid (AA), eicosapentaenoic acid (EPA) and docosahexaenoic acid (DHA) that are liberated from membrane phospholipids by phospholipases (PL)A_2_ upon cell stimulation [4,5]. Cyclooxygenases (COX)-1/2, six lipoxygenases (LOX) in humans, and CYP enzymes convert these free PUFA towards a broad spectrum of LM that act via specific receptors (mainly G protein-coupled receptors (GPCRs)). The most prominent LM related to inflammation are categorized into (i) the pro-inflammatory COX-derived prostaglandins (PGs) and 5-LOX-derived leukotrienes (LTs) [1,2,6] and (ii) the anti-inflammatory so-called specialized pro-resolving mediators (SPM) that are produced by multiple LOXs, COX-2 or CYP enzymes, with 15-LOX-1 as key enzyme [3,7,8]. SPM are potent endogenous immunoresolvents with beneficial functions in host defense, pain, organ protection, and tissue remodeling, possessing strong therapeutic potential for treatment of a multitude of inflammatory pathologies [9].

Rheumatoid arthritis (RA) is a chronic systemic autoimmune inflammatory disorder of the joints that results in bone and cartilage destruction [10], characterized by the production of eicosanoids, cytokines, adhesion molecules, and lymphocyte and macrophage infiltration in the synovium [11]. Current RA treatment options include disease-modifying anti-rheumatic drugs (DMARDs), corticosteroids, monoclonal antibodies and non-steroidal anti-inflammatory drugs (NSAIDs) [12], but there is still an unmet clinical need for novel RA treatments, ameliorating existing strategies. NSAIDs are the most widely used agents for symptomatic RA treatment which mainly act by blocking COX enzymes and thus the formation of pro-inflammatory PGs that contribute to the progressive destruction of cartilage and bone [13]. However, NSAIDs exert severe side effects and toxicity due to inhibition of PGs which hampers their clinical use [14]. In this respect, harnessing of SPM to promote resolution of inflammation is a new and alternative approach for the treatment of RA [15].

*Tripterygium wilfordii* Hook F (TwHF) is a traditional Chinese medicine made from the root of the plant that exhibits anti-inflammatory and immune-modulatory activities [16]. Among different TwHF preparations, *Tripterygium wilfordii* glycosides (TWG) tablets are the most widely clinically used form. A large number of clinical trials reported on the effectiveness and safety of TWG in RA [16,17,18], which was recently documented by a meta-analysis of 40 randomized controlled trials [19]. Some of these trials have even indicated that TWG may achieve better effectiveness than DMARDs monotherapy in patients with RA [20], but multi-organ toxicity and diverse side-effects of TWG have been reported [21]. The anti-rheumatic efficacy of TWG is based on immunosuppression, anti-inflammation, anti-angiogenesis, and bone and cartilage protection activities, due to reduced expression of proinflammatory cytokines and PGs, adhesion molecules and matrix metalloproteinases by macrophages, lymphocytes, synovial fibroblasts and chondrocytes [21,22], but the precise mechanisms of action are still obscure and await more detailed explorations. Triptolide and celastrol are the predominant constituents of TWG, accounting for the pharmacological features as well as for the reported toxicity [23]. Previous studies showed that TWG interferes with PGE_2_ biosynthesis by blocking the expression of COX-2 in a variety of cells [24,25], but how TWG impacts the complex LM networks and whether or not TWG affects SPM formation and LOX activities has not been addressed yet. We recently reported that celastrol is a direct 5-LOX inhibitor and promotes SPM biosynthesis [26]. Here we show that TWG significantly modulates the activity and the expression of various LM-biosynthetic enzymes leading to beneficial LM profiles supporting the anti-rheumatic properties of this remedy.

## 2. Materials and Methods

### 2.1. Materials

Tripterygium glycosides tablets (TWG, lei gong teng duogan pian, 10 mg/tablet, Approval number Z31020415) were obtained from Shanghai Fudan Fuhua Pharmaceutical Co., Ltd. (Shanghai, China). Zileuton (10006967), indomethacin (70270) and MK886 (21753), were supplied from Biomol GmbH (Hamburg, Germany); dexamethasone (CAS number 50-02-2) and ozagrel hydrochloride hydrate (CAS Number 78712-43-3) were purchased from Sigma-Aldrich (Steinheim, Germany).

Solvents for reversed phase-high performance liquid chromatography (RP-HPLC) of 5-LOX products were obtained from Merck (Darmstadt, Germany). Ultrapure water was produced by a Sartorius Arium 611 UV water purification system (Göttingen, Germany). Deuterated and non-deuterated LM standards for ultra-performance liquid chromatography-tandem mass spectrometry (UPLC-MS-MS) were purchased from Cayman Chemicals (Ann Arbor, MI, USA). All other chemicals and reagents were obtained from Sigma-Aldrich (Munich, Germany), unless stated otherwise.

### 2.2. High-Performance Liquid Chromatography (HPLC) Analysis of TWG and Celastrol

For analysis of TWG and celastrol determination we applied a gradient-based C18-reversed phase high-performance liquid chromatography (RP-HPLC) using an Agilent 1260 Infinity II system equipped with a diode-array-detection system (all Agilent Technologies Inc., Santa Clara, CA, USA) and a Xterra^®^ RP18 5 µm (4.6 mm × 250 mm) column (Waters Corporation, Milford, MA, USA). Detection was performed at 421 nm. The software OpenLAB CDS Rev. C.01.07 (Agilent Technologies) was used for method control and analysis. As mobile phase A acetonitrile (HPLC grade) and as mobile phase B 1% phosphoric acid in ultrapure water (both Carl Roth GmbH + Co. KG, Karlsruhe, Germany) were used. Each run was conducted with an injection volume of 50 µL. Elution was performed with 1 mL/min at 38 °C using the following gradient: 0–3 min 58A:42B, 3–8 min 58A:42B → 95A:5B, 8–13 min 95A:5B, 13–15 min 95A:5B → 58A:42B, 15–18 min 58A:42B. A calibration curve was established with celastrol (abcr GmbH, Karlsruhe, Germany) as reference substance in a concentration range from 0.5 to 250 µg/mL. Correlation coefficient displayed linearity with a value of 0.99997. The limit of detection (LOD) was 231.5 ng/mL, and the limit of quantification (LOQ) 241.7 ng/mL. One tablet containing 10 mg TWG was grinded, 1.5 mL acetonitrile were added and treated by ultrasonication. After centrifugation at 14,500 rpm for 5 min, the supernatant was analyzed by RP-HPLC. The amount of celastrol in the tablets containing 10 mg of TWG was determined at 15.8 µg or 0.158% based on the amount of extract, respectively. A representative chromatogram of TWG including detection of celastrol is shown in Supplementary Figure S1.

### 2.3. Isolation of Cells from Human Blood

For cell isolation, leukocyte concentrates from freshly withdrawn blood (16 IU heparin/mL blood) from healthy adult volunteers were obtained from the Department of Transfusion Medicine at the University Hospital of Jena, Germany. The experimental protocols were approved by the local ethical committee and were performed in accordance with guidelines and regulations; informed consent was obtained. Peripheral blood mononuclear cells (PBMC) were separated using dextran sedimentation, followed by centrifugation on lymphocyte separation medium (C-44010, Promocell, Heidelberg, Germany). PBMC were seeded in RPMI 1640 (Thermo Fisher Scientific, Schwerte, Germany) containing 10% (*v*/*v*) heat-inactivated fetal calf serum (FCS), 100 U/mL penicillin, and 100 mg/mL streptomycin in cell culture flasks (Greiner Bio-one, Frickenhausen, Germany) for 1.5 h at 37 °C and 5% CO_2_ for adherence of monocytes. Differentiation of monocytes to macrophages and polarization towards M1-monocyte-derived macrophages (MDM) and M2-MDM was performed as recently described [27]. Briefly, M0_GM-CSF_ and M0_M-CSF_ were generated by incubating monocytes with 20 ng/mL GM-CSF or M-CSF (Cell Guidance Systems Ltd., Cambridge, UK), respectively, for 6 days in RPMI 1640 supplemented with 10% FCS, 2 mmol/L L-glutamine and 100 U/mL penicillin-streptomycin. Then, 100 ng/mL LPS and 20 ng/mL IFNγ (Peprotech, Hamburg, Germany) were added to M0_GM-CSF_ for 48 h to obtain M1-MDM, while 20 ng/mL IL-4 (Peprotech) were added to M0_M-CSF_ for 48 h to obtain M2-MDM. To obtain polymorphonuclear leukocytes (PMNL), contaminating erythrocytes of pelleted PMNL after the density centrifugation step were removed by hypotonic lysis using water. PMNL were washed twice in ice-cold PBS and finally resuspended in PBS pH 7.4 containing 1 mg/mL glucose and then incubated as described below for the analysis of 5-LOX product formation. Platelet-rich plasma was obtained from the supernatants after density gradient centrifugation, mixed with PBS pH 5.9 (3:2 *v*/*v*), centrifuged (2100× *g*, 15 min, room temperature), and the pelleted platelets were resuspended in PBS pH 5.9/0.9% NaCl (1:1, *v*/*v*). Washed platelets were finally resuspended in PBS pH 7.4 and 1 mM CaCl_2_.

### 2.4. Determination of LM Formation in Human MDM

M2-MDM (2 × 10^6^/mL) were incubated with vehicle (0.1% DMSO) and TWG (3, 30 µg/mL) in PBS containing 1 mM CaCl_2_ for 180 min at 37 °C. The reaction was stopped by transferring supernatants (1 mL) into 2 mL ice-cold MeOH. In another set of experiments, with the scope to elucidate the effect of TWG on challenged M1-MDM, cells (2 × 10^6^/mL) were pre-treated with TWG (3, 30 µg/mL) in PBS containing 1 mM CaCl_2_ for 15 min prior to stimulation with 1% *Staphylococcus aureus* 6850 wt-conditioned medium (SACM) for another 90 min. Cultivation of *S. aureus* and preparation of SACM was performed as previously described [28]. The vehicle group received 0.1% DMSO. The reaction was stopped by transferring supernatants (1 mL) into 2 mL ice-cold MeOH.

To assess long-term effects of TWG on LM formation during macrophage polarization, M0_GM-CSF_ as well as M0_M-CSF_ (2 × 10^6^/mL) were pre-treated with TGW (1 µg/mL) for 15 min before the addition of LPS and IFNγ (for M1-MDM) or IL-4 (for M2-MDM) for subsequent 48 h polarization. Afterwards, 1% SACM was added to cells in PBS containing 1 mM CaCl_2_ for 90 min to induce LM biosynthesis. The reaction was stopped by transferring supernatants (1 mL) into 2 mL ice-cold MeOH. After addition of the deuterated LM standards (200 nM d8-5S-HETE, d4-LTB_4_, d5-LXA_4_, d5-RvD2, d4-PGE_2_ and 10 µM d8-AA; Cayman Chemical/Biomol GmbH, Hamburg, Germany), samples were processed for LM analysis using UPLC-MS-MS as described below.

### 2.5. Lipid Mediator Metabololipidomics by UPLC-MS-MS

Samples obtained from incubated MDM were kept at −20 °C for at least 60 min to allow protein precipitation. After centrifugation (1200× *g*, 4 °C, 10 min), acidified H_2_O (8 mL, final pH = 3.5) was added and samples were subjected to solid phase cartridges (Sep-Pak^®^ Vac 6cc 500 mg/6 mL C18; Waters, Milford, MA, USA). The columns had been equilibrated with 6 mL methanol and 2 mL H_2_O before sample loading. After washing with 6 mL H_2_O and then with 6 mL *n*-hexane, LM were eluted with 6 mL methyl formate. The samples were brought to dryness using an evaporation system (TurboVap LV, Biotage, Uppsala, Sweden) and resuspended in 100 µL methanol/water (50/50, *v*/*v*) for UPLC-MS-MS analysis. LM were analyzed with an Acquity™ UPLC system (Waters, Milford, MA, USA) and a QTRAP 5500 Mass Spectrometer (ABSciex, Darmstadt, Germany) equipped with a Turbo V™ Source and electrospray ionization. LM were eluted using an ACQUITY UPLC^®^ BEH C18 column (1.7 µm, 2.1 mm × 100 mm; Waters, Eschborn, Germany) at 50 °C with a flow rate of 0.3 mL/min and a mobile phase consisting of methanol-water-acetic acid of 42:58:0.01 (*v*/*v*/*v*) that was ramped to 86:14:0.01 (*v*/*v*/*v*) over 12.5 min and then to 98:2:0.01 (*v*/*v*/*v*) for 3 min [29]. The QTRAP 5500 was operated in negative ionization mode using scheduled multiple reaction monitoring (MRM) coupled with information-dependent acquisition. The scheduled MRM window was 60 s, optimized LM parameters were adopted [29], and the curtain gas pressure was set to 35 psi. The retention time and at least six diagnostic ions for each LM were confirmed by means of an external standard (Cayman Chemical/Biomol GmbH, Hamburg, Germany). Quantification was achieved by calibration curves for each LM. Linear calibration curves were obtained for each LM and gave *r*^2^ values of 0.998 or higher. Additionally, the limit of detection for each targeted LM was determined [29].

### 2.6. Determination of 5-LOX Activity in a Cell-Free Assay

Human recombinant 5-LOX was expressed in *E. coli* BL21 (DE3) transformed with pT3–5LO plasmid and purified by affinity chromatography on an ATP-agarose column as described previously [30]. Briefly, *E. coli* was lysed in 50 mM triethanolamine/HCl, pH 8.0, 5 mM EDTA, 60 μg/mL soybean trypsin inhibitor, 1 mM phenylmethanesulphonyl fluoride, 1 mM dithiothreitol and 1 mg/mL lysozyme and then sonified (3 × 15 s). The homogenate was then centrifuged at 40,000× *g* for 20 min at 4 °C. 5-LOX in the supernatant was partially purified by affinity chromatography on an ATP-agarose column (Sigma–Aldrich, Munich, Germany). Semi-purified 5-LOX was diluted in PBS containing EDTA (1 mM) and ATP (1 mM) and immediately used for 5-LOX activity assays.

Purified 5-LOX (0.5 µg) in PBS pH 7.4 containing EDTA (1 mM) and ATP (1 mM) was pre-incubated with vehicle (DMSO 0.1%), TWG (0.1, 0.3, 1, 3, 10 µg/mL) or zileuton (3 µM) for 15 min at 4 °C. Then, samples were pre-warmed for 30 s at 37 °C, and 2 mM CaCl_2_ plus 20 µM AA were added to start 5-LOX product formation. The reaction was stopped after 10 min by addition of one volume of ice-cold methanol, and the formed metabolites were analyzed by RP-HPLC. For the extraction of 5-LOX products, 200 ng of internal PGB_1_ standard was added to each sample and samples were centrifuged at 2000 rpm for 10 min. 5-LOX products were purified by solid phase extraction. After elution with 300 µL methanol, samples were analyzed for all-trans isomers of LTB_4_ and 5-H(p)ETE by RP-HPLC using a C-18 Radial-PAK column (Waters, Eschborn, Germany) as previously reported [31].

### 2.7. Determination of 5-LOX Activity in Human PMNL

Freshly isolated PMNL (5 × 10^6^) were pre-incubated with vehicle (DMSO, 0.1%), TWG (0.1, 0.3, 1, 3, 10 µg/mL), or zileuton (3 µM) for 10 min at 4 °C. Then, 2.5 µM A23187 was added together with 20 µM AA for another 10 min at 37 °C in order to induce 5-LOX product formation. The reaction was stopped by adding 1 mL of ice-cold MeOH to the cell suspension on ice. For the extraction of 5-LOX products (all-trans isomers of LTB_4_, LTB_4_ and 5-H(p)ETE), 200 ng of internal PGB_1_ standard was added, samples were centrifuged at 2000× *g* for 10 min, and solid phase extraction and analysis by RP-HPLC was performed as described above.

### 2.8. Determination of Cell Viability by MTT Assay

Freshly isolated human M0_GM-CSF_ or M0_M-CSF_ (2 × 10^5^/mL) in a 96-well plate were pre-incubated with 0.1% vehicle (DMSO), TWG (1, 3, 10, 30 µg/mL) or 1% Triton X-100 for 15 min, then LPS and IFNγ or IL-4 (for M1 and M2 polarization, respectively) were added for 48 h at 37 °C and 5% CO_2_. For short term treatment, polarized M1-MDM and M2-MDM were incubated with 0.1% vehicle (DMSO) or TWG (1, 3, 10, 30 µg/mL) or 1% Triton X-100 for 3 h. Then, calcein-AM and 3-(4,5-dimethylthiazol-2-yl)-2,5-diphenyltetrazolium bromide (MTT, 5 mg/mL, 20 µL; Sigma-Aldrich, Munich, Germany) solution were added in darkness for 2–3 h at 37 °C and 5% CO_2_. The formazan product was solubilized with sodium dodecyl sulfate (SDS, 10% in 20 mM HCl), and absorbance was measured at 570 nm using a Multiskan Spectrum microplate reader (Thermo Fisher Scientific, Schwerte, Germany).

### 2.9. Determination of COX-1 and -2 Activity

For determination of COX activities, purified ovine COX-1 (Cayman Chemicals; 50 units) or human recombinant COX-2 (Cayman Chemicals; 20 units) were diluted in Tris buffer (100 mM, pH 8) supplemented with glutathione (5 mM), EDTA (100 µM) and hemoglobin (5 µM). After pre-incubation with TWG, vehicle (0.1% DMSO) or indomethacin (IND, 10 µM) for 5 min at RT, the samples were pre-warmed for 30 s at 37 °C, and the reactions were started by addition of 5 µM AA (COX-1) or 2 µM AA (COX-2). After 5 min at 37 °C the reactions were stopped by addition of one volume of ice-cold methanol. Formation of COX-derived 12(*S*)-hydroxy-5-*cis*-8,10-*trans*-heptadecatrienoic acid (12-HHT) was analyzed by RP-HPLC on a Nova-Pak C18 Radial-Pak Column (4 µm, 5 × 100 mm, Waters) as described [31].

### 2.10. Determination of mPGES-1 Activity in a Cell-Free Assay

Microsomes of A549 cells stimulated by IL-1β (2 ng/mL for 48 h) were used as source for microsomal prostaglandin E_2_ synthase (mPGES)-1, as described elsewhere [32]. In brief, A549 cells were incubated with ice-cold homogenization buffer (0.1 M potassium phosphate buffer pH 7.4, 1 mM phenylmethanesulphonyl fluoride, 60 µg/mL soybean trypsin inhibitor, 1 µg/mL leupeptin, 2.5 mM glutathione and 250 mM sucrose). After sonication, the lysate was first centrifuged at 10,000× *g* for 10 min, and then at 174,000× *g* for 1 h at 4 °C. The pelleted microsomal fraction was then resuspended into 1 mL of homogenization buffer and diluted in a potassium phosphate buffer (0.1 M, pH 7.4) containing 2.5 mM glutathione. Afterwards, TWG, vehicle (0.1% DMSO), or the positive control MK886 (10 µM) were added for 15 min on ice in 100 µL incubation volume. The reaction was started after addition of PGH_2_ (20 µM) and stopped after 1 min at 4 °C using 100 µL of a stop solution (40 mM FeCl_3_, 80 mM citric acid, and 10 µM of 11β-PGE_2_ as internal standard). PGE_2_ and 11β-PGE_2_ were extracted by solid phase extraction using acetonitrile as eluent and quantified by RP-HPLC.

### 2.11. Determination of Thromboxane A Synthase Activity

Thromboxane A synthase (TXAS) activity was determined in lysates of human platelets. Freshly isolated cells were resuspended in ice-cold PBS pH 7.4 containing 1 mM EDTA (1 × 10^8^ cells/mL) and sonicated (4 × 10 s) on ice. The reaction was initiated by the addition of 20 µM PGH_2_ for 1 min at 4 °C in cell homogenates (1 mL) after pre-incubation with TWG (1 and 10 µg/mL), vehicle (0.1% DMSO) or the positive control ozagrel (OZA, 50 µM) for 15 min at 4 °C and terminated by the addition of ice-cold MeOH (2 mL). Then, samples were processed as described for LM metabololipidomics analysis and formed TXB_2_ was determined by UPLC-MS-MS, as reported above.

### 2.12. SDS-PAGE and Western Blot

M0_GM-CSF_ and M0_M-CSF_ MDM were treated with vehicle (DMSO), 1 µg/mL TWG or 100 nM dexamethasone for 15 min prior to addition of polarizing agents for 48 h at 37 °C and 5% CO_2_ and then lysed, as described previously [28]. Then, lysates were centrifuged (15,000 rpm, 5 min, 4 °C), cell supernatants were collected, and their protein concentration determined by DC-protein assay kit (Bio-Rad Laboratories GmbH, Munich, Germany). After addition of 4×SDS loading buffer (50 mM Tris-HCl, pH 6.8, 2% (*w*/*v*) SDS, 10% (*v*/*v*) glycerol, 1% (*v*/*v*) β-mercaptoethanol, 12.5 mM EDTA, 0.02% (*w*/*v*) bromophenol blue) to the lysates, samples were heated at 95 °C for 5 min. Equal aliquots were separated on 8% (for cPLA_2_α and COX-2), 10% (for 5-LOX, 15-LOX-1), 16% (for COX-1, mPGES-1) SDS-PAGE gels and then blotted onto nitrocellulose membranes (Amersham Protran Supported 0.45 μm nitrocellulose, GE Healthcare, Freiburg, Germany). The membranes were incubated with the following primary antibodies: rabbit polyclonal anti-cPLA_2_α,1:1000 (2832S; Cell Signaling Technology); rabbit polyclonal anti-5-LOX, 1:1000 (to a peptide corresponding to the C-terminal 12 amino acids of 5-LOX: CSPDRIPNSVAI; kindly provided by Dr. M. E. Newcomer, Louisiana State University, Baton Rouge, LA, USA); mouse monoclonal anti-15-LOX-1, 1:500 (ab119774; Abcam, Cambridge, UK); rabbit polyclonal anti-COX-1, 1:1000 (4841S; Cell Signaling Technology); rabbit monoclonal anti-COX-2, 1:1000 (12282S; Cell Signaling Technology); rabbit polyclonal anti-mPGES-1, 1:5000 (kindly provided by Dr. Per-Johan Jakobsson, Karolinska Institute, Stockholm, Sweden); mouse monoclonal anti-β-actin, 1:1000 (3700S; Cell Signaling). Immunoreactive bands were stained with IRDye 800CW Goat anti-Mouse IgG (H + L), 1:10,000 (926-32210, LI-COR Biosciences, Lincoln, NE), IRDye 800CW Goat anti-Rabbit IgG (H + L), 1:15,000 (926 32211, LI-COR Biosciences) and/or IRDye 680LT Goat anti-Mouse IgG (H + L), 1:40,000 (926-68020, LI-COR Biosciences), and visualized by an Odyssey infrared imager (LI-COR Biosciences, Lincoln, NE, USA). Data from densitometric analysis were background corrected.

### 2.13. Determination of Cytokine Levels

M0_M-CSF_ macrophages were treated with TWG (1 µg/mL), 100 nM dexamethasone or vehicle (0.1% DMSO) and stimulated with 100 ng/mL LPS for 20 h. For measurement of extracellular cytokine levels, supernatants were collected by centrifugation (2000× *g*, 4 °C, 10 min). The cytokines IL-1β and TNF-α were analyzed by in-house–made ELISA kits (R&D Systems, Bio-Techne, Abingdon, UK).

### 2.14. Flow Cytometry

M0_M-CSF_ MDM were treated with TWG (1 µg/mL) or vehicle (0.1% DMSO) for 48 h. Then, cells were stained in PBS pH 7.4 containing 0.5% BSA, 2 mM EDTA and 0.1% sodium azide by Zombie Aqua™ Fixable Viability Kit (Biolegend, San Diego, CA, USA) for 5 min at 4 °C to determine cell viability. Non-specific antibody binding was blocked by using mouse serum (10 min at 4 °C) prior to staining by the following fluorochrome-labelled antibodies (20 min, 4 °C): FITC anti-human CD14 (clone M5E2, #555397, BD Bioscience, San Jose, CA, USA), APC-H7 anti-human CD80 (clone L307.4, #561134, BD Bioscience), PE-Cy7 anti-human CD54 (clone HA58, #353115, Biolegend, Koblenz, Germany), PE anti-human CD163 (clone GHI/61, #556018, BD Biosciences, Heidelberg, Germany), APC anti-human CD206 (clone 19.2, #550889, BD Bioscience) to determine M1 and M2 surface marker expression using LSRFortessa^TM^ cell analyzer (BD Bioscience), and data were analyzed using FlowJo X Software (BD Bioscience).

### 2.15. Statistical Analysis

Results are expressed as mean + S.E.M. of each independent experiment, where *n* represents the indicated numbers from separate donors performed on different days. Statistical analysis and graphs were made by using GraphPad Prism 8 software (San Diego, CA, USA). Paired *t*-test was used to analyze experiments for comparison of two groups; while for multiple comparisons, ANOVA with Bonferroni or Dunnett multiple comparison tests were applied as indicated. A *p*-value ≤ 0.05 is a criterion for statistical significance.

## 3. Results

### 3.1. TWG Modulates LM Formation in Activated Pro-Inflammatory Macrophages

**Figure 1:**
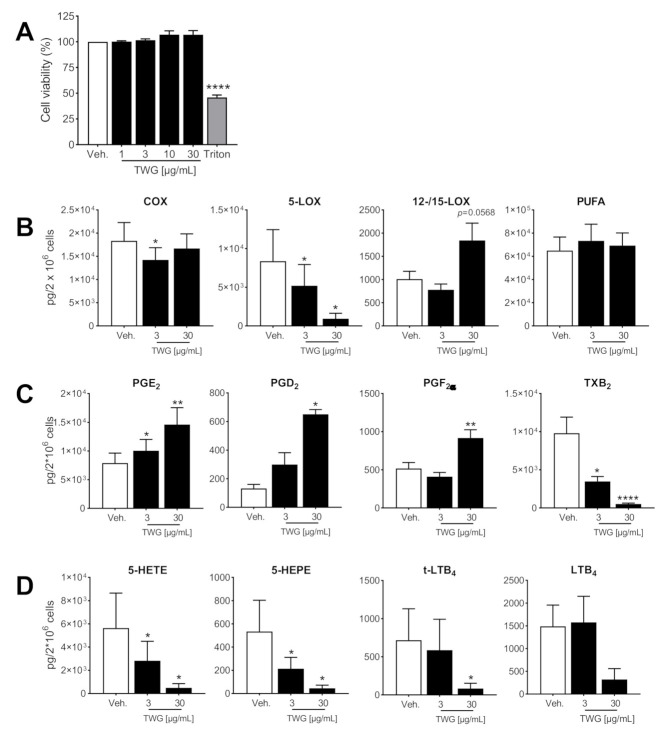
Effects of TWG on cell viability and LM modulation in human M1-MDM. (**A**) M1-MDM were treated with TWG at the indicated concentrations, 1% triton X-100, or 0.1% DMSO as vehicle for 3 h. Then, cell viability was assessed by MTT assay. Values are means + S.E.M., *n* = 3, expressed as percentage of vehicle control (=100%); **** *p* < 0.0001 TWG vs. control group, one-way ANOVA for multiple comparisons with Bonferroni’s correction. (**B**–**D**) M1-MDM were pre-treated with 3 and 30 µg/mL TWG or 0.1% DMSO as vehicle for 15 min and then stimulated with 1% *S. aureus*-conditioned medium (SACM) for 90 min. Produced LM were analyzed by UPLC-MS-MS in the supernatants. (**B**) The sum of COX products, 5-LOX products, 12/15-LOX products, and PUFA are expressed as pg/2 × 10^6^ cells of TWG-treated and vehicle-treated cells. (**C**,**D**) Individual members of COX products (**C**) and of 5-LOX products (**D**). Data are means ± S.E.M., *n* = 3 and were log-transformed for statistical analysis, * *p* < 0.05, ** *p* < 0.01, **** *p* < 0.0001, TWG vs. control group, one-way ANOVA and Dunnett’s multiple comparisons test.

Human MDM were polarized with IFNγ and LPS for 48 h towards a pro-inflammatory M1-like phenotype and preincubated with TWG (3 or 30 µg/mL, as low or high dose) for 15 min prior to activation with SACM [28] to induce LM biosynthesis. LM profile signatures in the medium were analyzed by UPLC-MS-MS after 90 min of these M1-MDM incubations, and revealed substantial amounts of COX- and 5-LOX-derived LM, with only minor formation of 12/15-LOX products (Table 1), as reported before [27,28]. Analysis of the cell viability by MTT assay revealed no detrimental effects of TWG within 180 min up to 30 µg/mL (Figure 1A). TWG at the high dose of 30 µg/mL potently suppressed formation of all 5-LOX products (LTB_4_, t-LTB_4_, 5-HETE, 5-HEPE and 7-HDHA) with minor efficiency at the low dose of 3 µg/mL (Table 1, Figure 1B,D). The sum of COX-derived products was not altered by TWG, however, PGE_2_ and PGD_2_ were elevated but formation of TXB_2_ was strongly diminished (Table 1, Figure 1B,C), suggesting that conversion of the COX product PGH_2_ by terminal prostanoid synthases is differentially affected by TWG. Of interest, TWG suppressed generation of 12-LOX products (i.e., 14-HDHA, 12-HETE and 12-HEPE) and RvD5, while 15-LOX products (i.e., 17-HDHA, 15-HETE and 15-HEPE) were rather elevated (Table 1), at least at 30 µg/mL TWG. The release of PUFA as LM substrates was not markedly affected by TWG.

### 3.2. TWG Selectively Inhibits 5-LOX among LM-Biosynthetic Enzymes

**Figure 2:**
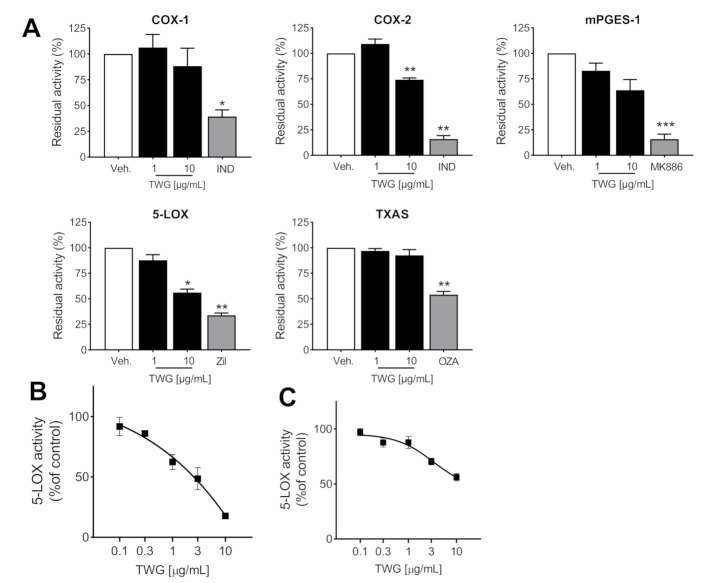
TWG selectively inhibits 5-LOX. (**A**) Effect of 1 and 10 µg/mL TWG on the residual activities of isolated ovine COX-1, human recombinant COX-2 and 5-LOX, mPGES-1 in microsomes from A549 cells, and TXAS in homogenates of human platelets. Data are expressed as percentage of vehicle (=100%) and given as means ± S.E.M., *n* = 3 (for COX-1/2, 5-LOX) or *n* = 4 (for mPGES1, TXAs). Indomethacin (IND) 10 µM, MK886 10 µM, and zileuton 3 µM were used as positive controls. * *p* < 0.05, ** *p* < 0.01, *** *p* < 0.001, TWG vs. control group, one-way ANOVA and Dunnett’s multiple comparisons test. (**B**,**C**) Concentration response curves of TWG for inhibition of (**B**) isolated human recombinant 5-LOX incubated with 20 µM AA, and (**C**) 5-LO in human intact PMNL stimulated with 2.5 µM A23187 plus 20 µM AA. Data are means + S.E.M., *n* = 3.

Intrigued by the significant and differential modulation of certain LM by TWG in activated MDM, we assessed the effects of TWG on enzymatic activities of isolated LM-biosynthetic enzymes in cell-free assays. TWG concentration-dependently inhibited the activity of human recombinant 5-LOX with IC_50_ of 2.9 µg/mL (Figure 2A,B). COX-1 activity was not inhibited, and COX-2 activity was moderately suppressed at 10 µg/mL TWG, the highest concentrations tested (Figure 2A). Surprisingly, TXAS was not affected despite efficient suppression of TXB_2_ formation in MDM (see Figure 1C), but mPGES-1 activity was reduced by 44% at 10 µg/mL TWG (Figure 2A). To explore 5-LOX inhibition we also assessed the effects of TWG in a robust and well-recognized cell-based assay, that is, A23187-activated human PMNL. Again, TWG concentration-dependently inhibited 5-LOX activity (Figure 2C), like in MDM.

### 3.3. TWG Induces the Formation of SPM and 12/15-LOX Products in Anti-Inflammatory Macrophages

**Figure 3:**
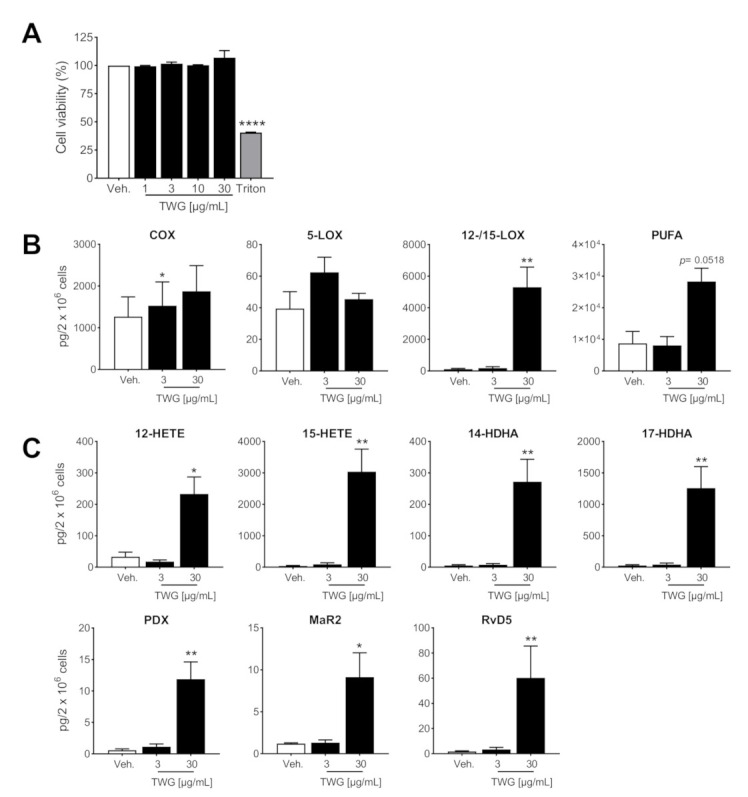
TWG induces LM production in anti-inflammatory human M2-MDM. (**A**) M2-MDM were treated with TWG at the indicated concentrations, 1% triton X-100, or 0.1% DMSO as vehicle for 3 h. Then, cell viability was assessed by MTT assay. Values are means + S.E.M., *n* = 3, expressed as percentage of vehicle control (=100%); **** *p* < 0.0001, TWG vs. control group, one-way ANOVA for multiple comparisons with Bonferroni’s correction. (**B**,**C**) M2-MDM were treated with 3 or 30 µg/mL TWG or with 0.1% DMSO as vehicle for 180 min and LM from cells supernatants were analyzed by UPLC-MS/MS. (**B**) The sum of COX products, 5-LOX products, 12/15-LOX products and PUFA are expressed as pg/2 × 10^6^ cells of TWG-treated and vehicle-treated cells. (**C**) Individual members of 12/15-LOX products, expressed as pg/2 × 10^6^ cells of TWG-treated and vehicle-treated cells. Data are means + S.E.M., *n* = 5 and were log-transformed for statistical analysis, * *p* < 0.05, ** *p* < 0.01, TWG vs. control group, one-way ANOVA and Dunnett’s multiple comparisons test.

Novel recent pharmacological approaches for treatment of chronic and excessive inflammatory disorders favor the formation of SPM, besides blocking pro-inflammatory PGs and LTs [8,33]. We employed the use of anti-inflammatory M2-MDM polarized with IL-4 that possess high capacities for SPM production due to substantial expression of 15-LOX-1, with moderate formation of PGs and LTs [27]. Like for M1-MDM, cell viability analysis by MTT assay showed no detrimental effects by TWG up to 30 µg/mL within 180 min for M2-MDM (Figure 3A). Intriguingly, exposure of M2-MDM to TWG at 30 µg/mL caused a massive formation of the SPMs PDX, RvD5 and MaR2 along with substantial formation of their monohydroxylated precursors 17-HDHA and 14-HDHA as well as other 12/15-LOX products with minor effects at 3 µg/mL TWG (Table 2, Figure 3B,C). The very low amounts of 5-LOX products in M2-MDM were not diminished by TWG (Table 2, Figure 3B). COX products were much less efficiently elevated by TWG as compared to SPM (Figure 3C), again with differential effects depending on the individual prostanoids: PGD_2_ was most potently elevated while TXB_2_ was not altered (Table 2). Together, these data indicate that TWG suppresses pro-inflammatory 5-LOX products in M1- but elevates inflammation-resolving SPM and 12/15-LOX products in M2-MDM.

### 3.4. TWG Modulates the Expression of LM-Biosynthetic Enzymes in Macrophages during Polarization

**Figure 4:**
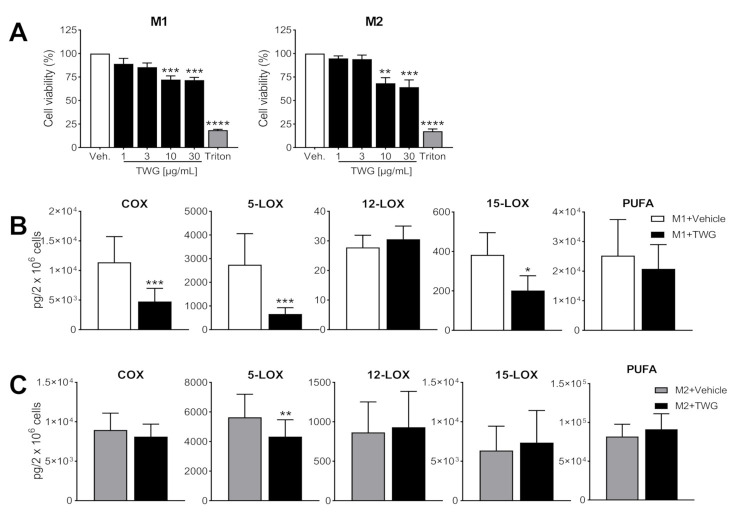
Effects of TWG on cell viability and LM pathway modulation during polarization of human M1- and M2-MDM. Human monocytes were differentiated towards M0 macrophages using GM-CSF or M-CSF for 6 days. These M0_GM-CSF_ and M0_M-CSF_ were pretreated for 15 min with test compounds or vehicle and then polarized for 48 h towards M1-MDM using LPS plus IFNγ or towards M2-MDM using IL-4, respectively. (**A**) TWG at the indicated concentrations, 10% triton X-100 or 0.1% DMSO as vehicle were added as test compounds and after 48 h polarization, cell viability was assessed by MTT assay. Data are means + S.E.M., *n* = 3 (both M1-MDM and M2-MDM), expressed as percentage of vehicle control (=100%); * *p* < 0.05, ** *p* < 0.01, *** *p* < 0.001, **** *p* < 0.0001, TWG vs. control group, one-way ANOVA for multiple comparisons with Bonferroni’s correction. (**B**,**C**) TWG (1 µg/mL) or 0.1% DMSO as vehicle was added. After 48 h polarization, M1-MDM (**B**) and M2-MDM (**C**) were incubated with 1% *S. aureus*-conditioned medium (SACM) for 90 min. Produced LM were analyzed by UPLC-MS-MS in the supernatants. The sum of COX products, 5-LOX products, 12/15-LOX products and PUFA are expressed as pg/2 × 10^6^ cells of TWG-treated and vehicle-treated cells. Data are means + S.E.M., *n* = 5 (both M1-MDM and M2-MDM). Data were log-transformed for statistical analysis, * *p* < 0.05, ** *p* < 0.01, *** *p* < 0.001, TWG vs. control group, paired *t*-test.

Next, we investigated if TWG may also affect LM biosynthetic pathways during polarization of the MDM with consequence for LM-producing capacities of the cells. MDM were pre-treated with TWG for 15 min, polarized for 48 h towards M1-MDM using IFNγ plus LPS or towards M2-MDM using IL-4, harvested, and then activated with SACM to elicit LM production. Analysis by MTT assay showed that during 48 h exposure to TWG, both M1- and M2-MDM were susceptible to TWG ≥ 3 µg/mL with loss of viability (Figure 4A), and thus we applied lower TWG concentrations of only 1 µg/mL. As shown in Table 3 and Figure 4B, 5-LOX and COX products formed by M1-MDM were significantly impaired when cells had been pre-treated with TWG, and also 15-LOX products were diminished, while 12-LOX products remained elevated; release of PUFA was not markedly altered. In M2-MDM, a significant but moderate reduction of 5-LOX products was observed without significant alterations of other LM (Table 3, Figure 4C).

**Figure 5:**
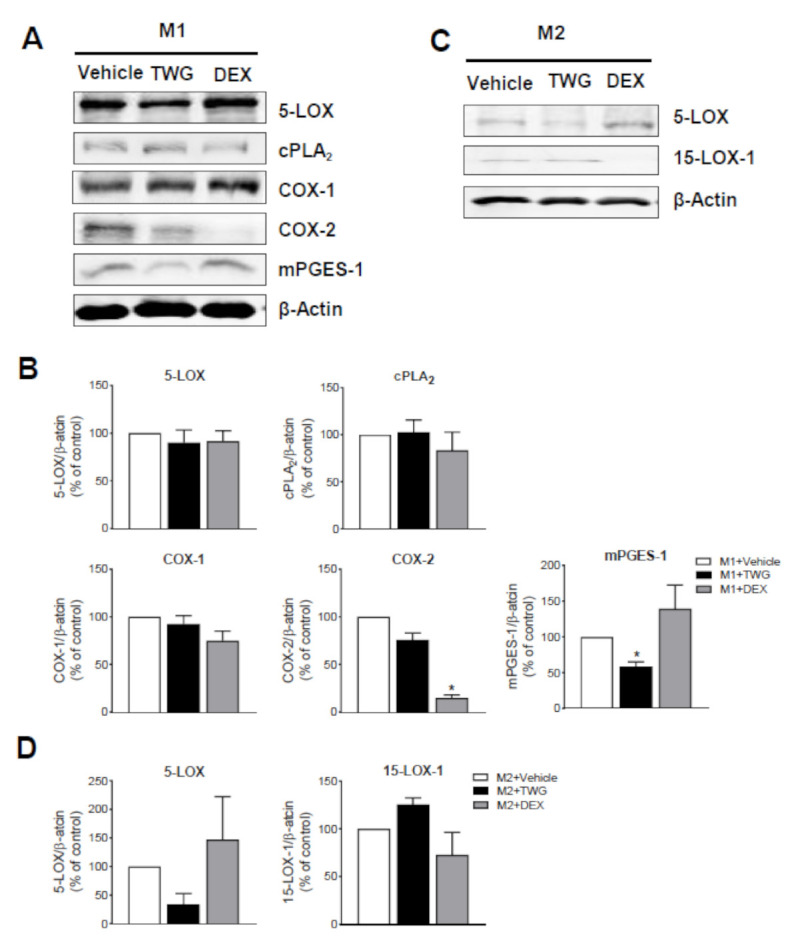
Effects of TWG on LM-biosynthetic enzyme expression during polarization of human M1- and M2-MDM. Human monocytes were differentiated towards M0 macrophages using GM-CSF or M-CSF for 6 days. These M0_GM-CSF_ and M0_M-CSF_ were pretreated for 15 min with 1 µg/mL TWG, 100 nM dexamethasone (DEX) or 0.1% DMSO as vehicle and then polarized for 48 h towards M1-MDM using LPS plus IFNγ or towards M2-MDM using IL-4, respectively. (**A**–**D**) Protein expression in lysates of M1-MDM (**A**,**B**) and M2-MDM (**C**,**D**) were analyzed by Western blotting. For densitometric analysis (**B**,**D**), 5-LOX, cPLA_2_, COX-1, COX-2 and mPGES-1 proteins were normalized to β-actin; exemplary results (**A**,**C**) are shown as means + S.E.M. from *n* = 3 separate donors (both M1-MDM and M2-MDM). Values shown are percentages of DMSO controls (=100%). Densitometric ratios were used for statistical analysis, * *p* < 0.05, TWG vs. control group, one-way ANOVA and Dunnett’s multiple comparisons test.

We then assessed if TWG affects the amounts of LM-biosynthetic enzymes during polarization; dexamethasone (DEX, 100 nM) was used as reference drug. Western blot analysis with M1-MDM showed no significant changes of cPLA_2_, 5-LOX, COX-1 and COX-2 protein amounts by TWG, but mPGES-1 was significantly reduced; DEX suppressed COX-2 protein levels as expected without affecting other enzymes addressed (Figure 5A,B). In M2-MDM, TWG impaired the amounts of 5-LOX protein (yet no statistical significance was reached) without affecting 15-LOX-1 levels, while DEX caused the opposite: it slightly decreased the protein amounts of 15-LOX-1 without affecting those of 5-LOX (Figure 5C,D).

### 3.5. TWG Suppresses Pro-Inflammatory Cytokines and Impacts Macrophage Polarization

**Figure 6:**
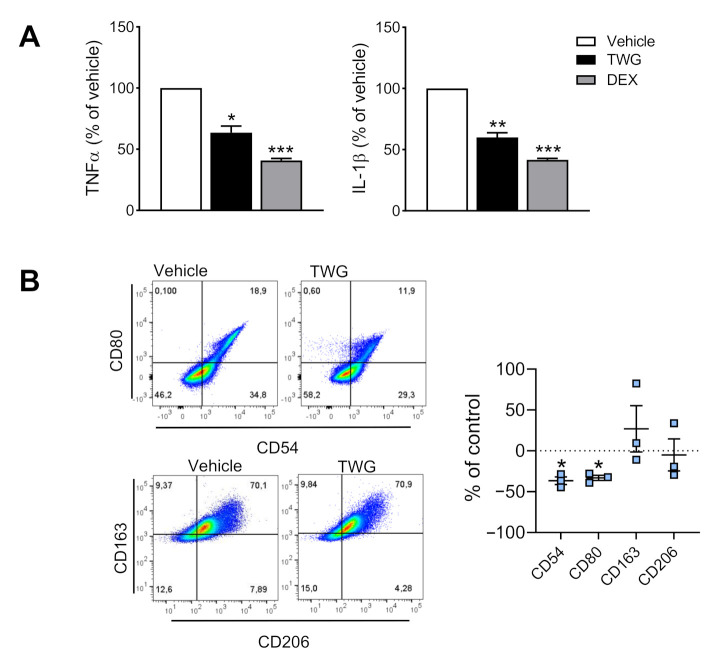
Effects of TWG on cytokine release and macrophage surface marker expression. Human monocytes were differentiated towards M0 macrophages using M-CSF for 6 days. (**A**) The M0_M-CSF_ were then treated with 1 µg/mL TWG, 100 nM dexamethasone (DEX) or 0.1% DMSO as vehicle. After 20 h of stimulation with 100 ng/mL LPS, TNFα and IL-1β in the cell supernatants were analyzed by ELISA (presented as 100% of vehicle). Data are means + S.E.M., *n* = 4 separate experiments. Absolute values (shown in pg/10^6^ cells) were log-transformed for statistical analysis, * *p* < 0.05, ** *p* < 0.01, *** *p* < 0.001, TWG vs. control group, one-way ANOVA and Dunnett’s multiple comparisons test. (**B**) The M0_M-CSF_ were then treated with 1 µg/mL TWG or 0.1% DMSO as vehicle. After 48 h, expression of the surface markers CD54 and CD80 (M1-like) as well as CD163 and CD206 (M2-like) among living CD14^+^ cells was analyzed by flow cytometry; shown are representative pseudocolor dot plots of M1-like and M2-like surface markers. Mean fluorescence intensity (MFI) of each marker was determined. The change of the MFI from TWG-treated macrophages against the MFI of DMSO-treated cells (control) was calculated and given in % of control in scatter dot plots as single values and means ± S.E.M., *n* = 3. Statistics are calculated with raw data (MFI), * *p* < 0.05 TWG vs. control group, ratio paired *t*-test.

Since TWG suppressed pro-inflammatory COX and 5-LOX pathways during polarization of M1-MDM, we studied if also pro-inflammatory cytokines are affected by TWG. In fact, TWG (1 µg/mL) blocked release of TNF-α and IL-1β in M0_M-CSF_ MDM, similar as the reference drug DEX (Figure 6A). Finally, we investigated if TWG may affect macrophage polarization by assessing CD54 and CD80 as M1 markers and CD163 and CD206 as markers for M2 macrophages [27]. In agreement with the impaired capacities to generate pro-inflammatory LM (i.e., COX/5-LOX products) and cytokines (TNF-α and IL-1β) but increasing anti-inflammatory SPM, the markers for M1-MDM, i.e., CD54 and CD80 were significantly decreased but those for M2 (CD163, CD206) were slightly increased (Figure 6B). Together, TWG apparently impairs polarization of pro-inflammatory macrophages, characterized by suppression of pro-inflammatory cytokines and LM in M1, while promoting inflammation-resolving SPM in M2 macrophages.

## 4. Discussion

Employing a comprehensive LM metabololipidomics approach we show here that TWG beneficially modulates the biosynthesis of LM networks in various innate immune cells, that is, suppressing the formation of pro-inflammatory 5-LOX products and thromboxane in M1-MDM and PMNL, but elevating the levels of inflammation-resolving SPM in anti-inflammatory M2-MDM. Our results suggest multiple points of attack of TWG in the LM networks, such as direct inhibition of 5-LOX activity and blocking 5-LOX expression, reducing the expression and activity of mPGES-1, and intriguingly, induction of the activation of 12/15-LOXs. Since these bioactions of TWG occurred at fairly low effective concentrations (i.e., 1–30 µg/mL) that might be of pharmacological relevance, our data suggest that such beneficial switch from pro-inflammatory to pro-resolving LM production in innate immune cells may contribute to the anti-rheumatic features of TWG documented in numerous clinical trials of RA [16,18,19,21]. Future studies on how TWG modulates LM production in co-culture system that more closely recapitulate RA pathology such as human RA synovial fibroblasts or synovial cell lines with macrophages may sustain the direct link with RA.

Modulation of LM networks in innate immune cells by TWG has not been reported yet, to the best of our knowledge. Only one study was published that demonstrated suppression of PGE_2_ formation due to inhibition of COX-2 expression [24]. Therefore, modulation of cellular LOX activities and thus LT and SPM formation by TWG are novel findings. We confirmed PGE_2_ suppression using pro-inflammatory human M1-MDM that acquire substantial amounts of COX-2 protein during polarization [27]. When TWG was present during M1-MDM polarization, the cells showed reduced capacities to generate PGE_2_, but also other COX-derived prostanoids such as PGD_2_, PGF_2_α and TXB_2_ upon subsequent stimulation. Interestingly, we discovered that besides interference with COX-2, TWG also inhibited the induction of mPGES-1 protein and slightly impaired its enzymatic activity, which may sustain the reduced PGE_2_ formation by TWG. Among the three PGE_2_ synthases, mPGES-1 is an inducible isoform and strongly linked to inflammatory diseases [34], including RA [35].

While long-term treatment of M1-MDM with TWG clearly impaired formation of all COX-derived products possibly due to COX-2 suppression, short term exposure of these cells to TWG selectively blocked formation of only TXB_2_, suggesting an inhibitory effect on the biosynthetic branch from PGH_2_ towards TXA_2_ [36] potentially by acting on TXAS. Surprisingly, TWG however failed to inhibit TXAS in a cell-free assay, excluding direct interaction of TWG with the enzyme. It is reasonable that TXAS inhibition requires the intracellular environment, for example to convert the responsible bioactive ingredient(s) into the active form, a phenomenon well-known for naturally occurring quinones (like celastrol), which act as potent inhibitors of 5-LOX as reduced hydroquinones [37].

Our study reveals 5-LOX as a direct target of TWG by using cell-free assays and inhibition of 5-LOX products in M1-MDM and in PMNL; 5-LOX and its products, especially LTB_4_, have been implicated in RA as well [6,11], playing essential roles in the induction of pain and bone damage [38]. Also, 5-LOX was strongly expressed in lining and sublining macrophages, neutrophils and mast cells of RA synovial biopsies which was suppressed by glucocorticoid treatment [39]. Results of several studies underline the crucial role for LTB_4_ and its receptor BLT1 in the pathogenesis of inflammatory arthritis [40]. Therefore, the potent impairment of LT formation in pro-inflammatory M1-MDM and PMNL due to 5-LOX inhibition may reasonably contribute to amelioration of RA by TWG treatment. This is also supported by our recent finding that the pentacyclic triterpenoid quinone methide celastrol, as major bioactive TWG constituent [23], potently inhibits 5-LOX in cell-free and cell-based assays at 0.1 to 1 µM [26]. Our RP-HPLC analysis and calculation revealed a celastrol content of 0.158% in TWG, implying that at the IC_50_ of 2.9 µg/mL TWG for 5-LOX, 0.0044 µg/mL or ~0.1 µM celastrol is present, which fits to the reported IC_50_ of 0.19 µM for celastrol under the same 5-LOX assay conditions [26].

Although our results are in favor of beneficial properties of TWG for treatment of RA, the well-known toxicity of TWG observed in clinical trials is still a concern [18,19]. We have carefully considered the issue of potential cytotoxicity and thus avoided the use of high concentrations of TWG, i.e., ≤30 µg/mL in short-term and 1 µg/mL in long-term incubations, along with exclusion of cytotoxic effects under the experimental conditions that we employed.

We suggest that promoting a switch from pro-inflammatory LTs and PGs towards inflammation-resolving SPM by smart manipulation of LM networks using TWG might a beneficial strategy for RA treatment. In contrast to LTs and PGs that initialize and maintain persistent and excessive inflammation-promoting various inflammatory pathologies [1,2], the SPM are anti-inflammatory immunoresolvents and promote the resolution of inflammation leading to tissue repair and return to homeostasis [3,9,41]. Recent studies indicated that RA may arise from a decreased ability of the immune response to engage resolution programs that terminate inflammation and prevent chronicity [15]. Experimental models of delayed or non-resolving joint inflammation showed that SPM, i.e., RvD3, were downregulated [42]. In arthritic patients, synovial levels of RvE2 correlated with decreased joint pain [43]. Recently, SPM levels in peripheral blood of RA patients were linked with both DMARD responsiveness and disease pathotype [44] and strategies to increase SPM production have been shown to be connected with decreased joint inflammation and promotion of joint protection [45]. Intriguingly, exposure of M2-MDM to 30 µg/mL TWG caused massive induction of 12-/15-LOX product formation including the biosynthesis of SPM. Note that in contrast to reduced capacities of MDM to generate COX and 5-LOX products upon long-term treatment with TWG, the remedy did not impair the ability to form 12/15-LOX-derived LM. How such potent 12-/15-LOX activation is induced by TWG remains to be investigated but might be again caused by celastrol that induced SPM formation in M2-MDM at 1 µM [26], the calculated celastrol concentration present in 30 µg/mL TWG.

Besides celastrol, other bioactive ingredient(s) contained in TWG might be responsible for the observed actions on LM pathways, especially related to the suppression of the expression of the LM biosynthetic enzymes COX-2, mPGES-1 and 5-LOX. The diterpenoid triepoxide triptolide is another major constituent of TwHF with glucocorticoid-like properties that mediates many of the pharmacological actions and the anti-rheumatic activity of TWG [23]. Studies on celastrol and triptolide with focus on modulation of expression of LM pathways under long-term conditions are currently ongoing in our laboratory.

Taken together, TWG causes beneficial modulation of LM biosynthesis in prime innate immune cells by suppressing pro-inflammatory PG and LT pathways via multiple points of attack and by promoting SPM formation. In view of the well-established detrimental impact of PG and LT in RA pathology on one hand and the beneficial features of SPM on the other, our findings may help to explain the ameliorating effects of TWG in RA treatment and encourage future evaluation of the efficacy and safety of such remedies.

## Figures and Tables

**Table 1:** Heatmap of LM produced by pro-inflammatory human M1-MDM and effects of Table 1**.** MDM were pre-treated for 15 min with 3 or 30 µg/mL TWG or 0.1% DMSO as vehicle. Then, cells were stimulated with 1% *S. aureus*-conditioned medium (SACM) for 90 min. LM were analyzed by UPLC-MS/MS in the supernatants of cells. Data are means ± S.E.M., *n* = 3.

								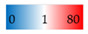			
LM	Vehicle			TWG, 3 µg/mL			%	TWG, 30 µg/mL			%
PGE_2_	7899	±	1734	10,047	±	1975	**127**	14,612	±	2939	**185**
PGD_2_	132	±	28	300	±	82	**226**	651	±	33	**492**
PGF_2α_	517	±	78	410	±	56	**79**	917	±	109	**177**
TXB_2_	9799	±	2114	3474	±	636	**35**	517	±	119	**5**
11-HETE	859	±	215	560	±	119	**65**	1265	±	323	**147**
11-HEPE	37	±	7	26	±	2	**69**	54	±	12	**145**
5-HETE	5643	±	3000	2825	±	1677	**50**	492	±	364	**9**
5-HEPE	534	±	269	214	±	97	**40**	46	±	27	**9**
t-LTB_4_	717	±	413	587	±	405	**82**	84	±	69	**12**
LTB_4_	1488	±	467	1577	±	572	**106**	322	±	237	**22**
7-HDHA	55	±	11	21	±	6	**38**	9	±	2	**16**
17-HDHA	79	±	14	72	±	12	**91**	96	±	11	**122**
15-HETE	821	±	156	630	±	101	**77**	1649	±	392	**201**
15-HEPE	13	±	2	11	±	1	**86**	26	±	1	**202**
14-HDHA	14	±	4	12	±	4	**87**	10	±	3	**73**
12-HETE	75	±	22	49	±	26	**66**	56	±	29	**74**
12-HEPE	8	±	2	5	±	2	**65**	5	±	2	**69**
RvD5	3	±	0	2	±	0	**65**	1	±	0	**39**
AA	35,272	±	6967	40,740	±	7838	**116**	40,043	±	7620	**114**
EPA	4272	±	659	5274	±	685	**123**	4669	±	575	**109**
DHA	25,373	±	4809	27,362	±	6445	**108**	24,547	±	3921	**97**

**Table 2:** Heatmap of LM produced by anti-inflammatory human M2-MDM and effects of TWG. M2-MDM were treated for 180 min with 3 or 30 µg/mL TWG or 0.1% DMSO as vehicle. Then, formed LM released into the medium were extracted and analyzed by UPLC-MS/MS. Data are means ± S.E.M., *n* = 5.

								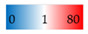			
LM	Vehicle			TWG, 3 µg/ml			Fold	TWG, 30 µg/ml			Fold
PGE_2_	34	±	7	46	±	9	**1.4**	121	±	28	**3.6**
PGD_2_	9	±	3	35	±	2	**3.7**	217	±	52	**22.8**
PGF_2α_	41	±	16	61	±	21	**1.5**	131	±	21	**3.2**
TXB_2_	1181	±	454	1386	±	546	**1.2**	1409	±	632	**1.2**
11-HETE	5	±	2	6	±	2	**1.2**	44	±	10	**8.5**
11-HEPE	3	±	1	2	±	1	**0.8**	9	±	1	**3.4**
5-HETE	25	±	8	46	±	11	**1.8**	30	±	2	**1.2**
5-HEPE	5	±	1	7	±	1	**1.3**	5	±	1	**1.0**
t-LTB_4_	6	±	2	6	±	2	**1.0**	6	±	0	**1.0**
LTB_4_	3	±	1	4	±	1	**1.1**	5	±	1	**1.5**
7-HDHA	12	±	2	13	±	3	**1.1**	18	±	3	**1.5**
17-HDHA	27	±	11	43	±	23	**1.6**	1259	±	340	**46.3**
15-HETE	38	±	15	92	±	44	**2.4**	3039	±	716	**78.9**
15-HEPE	10	±	3	20	±	10	**2.1**	431	±	92	**45.0**
14-HDHA	6	±	2	8	±	4	**1.4**	272	±	71	**49.2**
12-HETE	33	±	14	18	±	5	**0.5**	233	±	54	**7.0**
12-HEPE	4	±	1	4	±	2	**1.0**	70	±	15	**18.3**
PDX	1	±	0	1	±	0	**2.0**	12	±	3	**20.5**
RvD5	2	±	1	3	±	2	**2.1**	60	±	25	**38.3**
MaR2	1	±	0	1	±	0	**1.1**	9	±	3	**7.6**
AA	4155	±	1678	4330	±	1388	**1.0**	15,991	±	1863	**3.8**
EPA	680	±	297	546	±	189	**0.8**	3148	±	245	**4.6**
DHA	3926	±	1814	3232	±	1294	**0.8**	9167	±	2329	**2.3**

**Table 3:** Heatmap of LM produced by human M1- and M2-MDM and effects of TWG. M0_GM-CSF_ and M0_M-CSF_ MDM were pre-treated with 1 µg/mL TWG or 0.1% DMSO as vehicle for 15 min prior to polarization toward M1- or M2-MDM for 48 h by the addition of LPS/IFNγ (M1-MDM, left) and IL-4 (M2-MDM, right), respectively. Cells in PBS plus 1 mM CaCl_2_ were then stimulated with 1% SACM for 90 min. LM from cells released into the supernatants were extracted and analyzed by UPLC-MS/MS. Data are means ± S.E.M., *n* = 5 (both M1-MDM and M2-MDM); n.d., not detectable.

				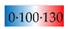								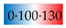			
LM (M1)	Vehicle			TWG			%	LM (M2)	Vehicle			TWG			%
PGE_2_	5670	±	2179	1961	±	986	**35**	PGE_2_	650	±	123	774	±	158	**119**
PGD_2_	90	±	30	39	±	14	**43**	PGD_2_	85	±	19	107	±	26	**126**
PGF_2α_	303	±	81	155	±	51	**51**	PGF_2α_	238	±	70	259	±	93	**109**
TXB_2_	5326	±	2078	2622	±	1158	**49**	TXB_2_	7994	±	2012	6988	±	1382	**87**
11-HETE	288	±	116	107	±	49	**37**	11-HETE	279	±	54	288	±	63	**103**
11-HEPE	17	±	4	8	±	3	**48**	11-HEPE	24	±	5	27	±	8	**113**
5-HETE	1346	±	635	353	±	134	**26**	5-HETE	3886	±	1074	3008	±	805	**77**
5-HEPE	142	±	67	42	±	14	**29**	5-HEPE	483	±	153	411	±	130	**85**
t-LTB_4_	378	±	204	74	±	34	**20**	t-LTB_4_	310	±	74	222	±	36	**72**
LTB_4_	880	±	406	194	±	81	**22**	LTB_4_	967	±	301	695	±	199	**72**
7-HDHA	30	±	10	14	±	3	**47**	7-HDHA	59	±	10	44	±	7	**74**
17-HDHA	56	±	12	45	±	16	**81**	17-HDHA	792	±	205	802	±	266	**101**
15-HETE	319	±	99	150	±	58	**47**	15-HETE	5070	±	2588	5922	±	3409	**117**
15-HEPE	9	±	1	7	±	2	**75**	15-HEPE	510	±	282	642	±	397	**126**
14-HDHA	7	±	2	9	±	3	**128**	14-HDHA	211	±	79	214	±	77	**101**
12-HETE	18	±	2	18	±	2	**102**	12-HETE	560	±	259	606	±	314	**108**
12-HEPE	3	±	0	3	±	1	**114**	12-HEPE	96	±	49	112	±	63	**117**
PDX	n.d.			n.d.			-	PDX	2	±	1	2	±	1	**83**
RvD5	n.d.			n.d.			-	RvD5	25	±	8	19	±	7	**73**
AA	12,388	±	6118	9969	±	4046	**80**	AA	51,707	±	10,270	57,824	±	12,417	**112**
EPA	1926	±	674	1499	±	343	**78**	EPA	11,310	±	2601	13,841	±	3753	**122**
DHA	10,974	±	5412	9362	±	3783	**85**	DHA	18,926	±	3274	19,709	±	3947	**104**

## Data Availability

The data presented in this study are available on reasonable request from the corresponding author. The data are not publicly available due to privacy.

## Supplementary Materials

The following are available online at https://www.mdpi.com/article/10.3390/biom11050746/s1: Supplementary Figure S1: Chromatograms of the RP-HPLC analysis of TWG and determination of celastrol.
