# Supplementary material for: Beneficial Modulation of Lipid Mediator Biosynthesis in Innate Immune Cells by Antirheumatic Tripterygium wilfordii Glycosides
Source: Biomolecules. 2021 May 17;11(5):746. doi: 10.3390/biom11050746 (PMC8155965; doi:10.3390/biom11050746)
Supplement: Supplementary file 1 [file biomolecules-11-00746-s001.zip › biomolecules-1203255-supplementary.pdf]

Favorable modulation of lipid mediator biosynthesis in innate immune cells by antirheumatic *Tripterygium wilfordii* glycosides

Kehong Zhang[1,2,§], Simona Pace[1,§], Paul M. Jordan[1], Lukas Klaus Peltner[1], Alexander Weber[3], Dagmar Fischer[3], Robert Klaus Hofstetter[1], Xinchun Chen[2], Oliver Werz[1,*]

[1]Department of Pharmaceutical/Medicinal Chemistry, Institute of Pharmacy, Friedrich-Schiller-University, Philosophenweg 14, D-07743 Jena, Germany.

[2]Guangdong Provincial Key Laboratory of Regional Immunity and Diseases, Department of Pathogen Biology, Shenzhen University School of Medicine, Shenzhen 518000, China.

[3]Department of Chemistry and Pharmacy, Pharmaceutical Technology, Friedrich-Alexander-Universität Erlangen-Nürnberg, Cauerstrasse 4, 91058 Erlangen, Germany.

Supplemental Material

Supplementary Figure S1

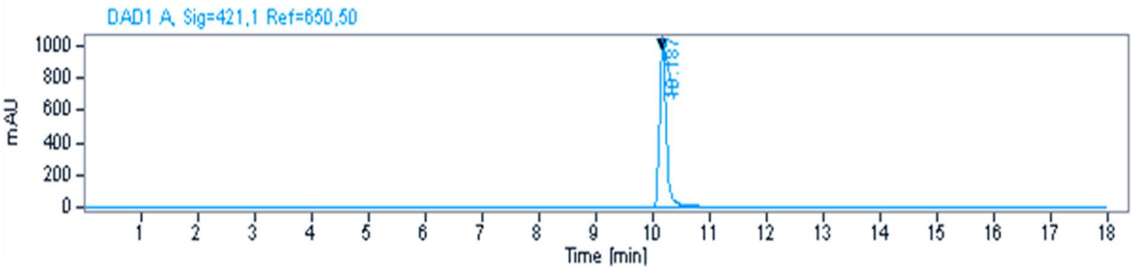

(**A**)

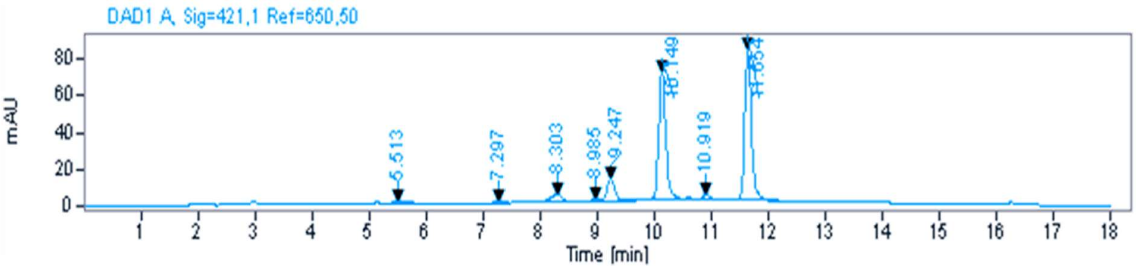

(**B**)

Figure S1 RP-HPLC Chromatograms of TWG and determination of celastrol

Chromatogram of celastrol reference substance (**A**) and of TWG (**B**) at 421 nm VIS detection. Under optimized conditions retention times of 10.168 ± 0.02 min for celastrol could be realized.
